# Detection of EBV in reactive and neoplastic lymphoproliferations in adults—when and how?

**DOI:** 10.1007/s12308-014-0209-0

**Published:** 2014-07-03

**Authors:** Christiane Stuhlmann-Laeisz, Ilske Oschlies, Wolfram Klapper

**Affiliations:** Hematopathology Section and Lymph Node Registry Kiel, University Hospital Schleswig-Holstein, Arnold-Heller-Straße 3, Haus 14, 24105 Kiel, Germany

**Keywords:** Lymphoma, Latency type, ENBA2, EBER, LMP1

## Abstract

Lymphoproliferations associated with Epstein–Barr Virus (EBV) in adult patients pose a diagnostic challenge for pathologists for several reasons. First, the EBV lymphoproliferations represent a clinically and histologically very broad spectrum ranging from self-limiting lymphoproliferations to manifest malignant lymphomas. Second, the classification of these diseases is not solely based on histopathology but rather requires a synopsis of clinical as well as pathological features. And third, a resource-efficient diagnostic procedure demands a deliberate strategy for selecting the tissue specimens that are to be tested for EBV. We describe how the clinical context and histological features may indicate to histopathologists which lymphatic tissues should be tested for the presence of EBV and how these features guide the classification. We provide recommendations as to which biopsy specimens should be investigated for EBV and which methods for detecting viral association are appropriate.

## Introduction

The Epstein–Barr virus (EBV) was described by Michael Anthony Epstein and Yvonne Barr, who isolated EBV virus particles from endemic Burkitt lymphoma specimens [1]. EBV is a double stranded DNA virus of the herpes virus family and is the most common virus affecting humans. Exposure to EBV usually occurs during childhood. The infection occurs through oral transmission via saliva. By the age of 40, over 90 % of the world population has experienced an EBV infection. EBV also plays a pathogenic role, mainly in the development of several T cell and B cell lymphoproliferations (see below) but is also associated with different types of rare epithelial and mesenchymal neoplasms [2].

EBV is mainly a B-lymphotropic and epitheliotropic virus and rarely infects T cells and NK cells. The life cycle of EBV is composed of a lytic state and a latency state, the latter allowing lifelong persistence of the virus in the host. The replication cycle of EBV is composed of three phases: (i) entry into either epithelial cells or naive B cells after oropharyngeal transmission, (ii) lytic replication, and (iii) latency. Once a cell has become infected, the viral capsid dissolves and the DNA is transported into the cell nucleus. In epithelial cells, the entry of EBV into the cell is usually directly followed by the lytic replication of the virus, which leads to production of infectious virions using the virus’s own replication machinery. EBV persists in B cells throughout the differentiation from naive B cells to germinal center cells to long-lived memory B cells [2]. In memory B cells, no infectious virions are produced in the latency state. Dependent on the expression of specific EBV-associated proteins and RNAs, three different viral latency types have been described, which can be reproduced to a certain extent by immunohistochemical markers [2] (Table 1).

**Table 1:** EBV-latency types and expressed antigens

Latency type	EBV-associated antigens
0/I	EBER, EBNA1
II (default program)	EBER, EBNA1, LMP1, LMP2B
III (growth program)	EBER, EBNA1-6, LMP1, LMP2A + B
lytic	all lytic antigens (e.g., ZEBRA)

## Primary infection by EBV—clinical and histological features

Most humans become infected with EBV during childhood, when the infection usually proceeds asymptomatically or with mild symptoms that are undistinguishable from other viral infections. If the infection occurs in young adults and adolescents, it can manifest as mononucleosis (glandular fever). Primary EBV infection in adult patients is rare. Histologically, pimary infection manifests in childhood or young adulthood as ulcerative tonsillitis. Only occasionally are lymph nodes or other sites affected. EBV tonsillitis differs from unspecific tonsillitis related to other causes in its histologically detectable polymorphic lymphoid infiltration with abundant plasmablasts and occasional Hodgkin-like cells expanding in the interfollicular area of the tonsils. In affected lymph nodes, an extension of the interfollicular areas with a similarly polymorphic infiltration to that in tonsils can be observed. Within the peripheral blood and bone marrow, CD8 positive lymphoblasts (Pfeiffer cells) might be observed. The histomorphological picture of primary EBV infection shares histomorphological features with other EBV-driven lymphoproliferative diseases (LPD, Table 2). Therefore, the definite diagnosis of infectious mononucleosis is based on the following features: (i) young patient age, (ii) sole or main manifestation in the tonsils, (iii) absence of immunosuppression in the patient, and most importantly (iv) a serological test indicating EBV primary infection (positive IgM titer).

**Table 2:**
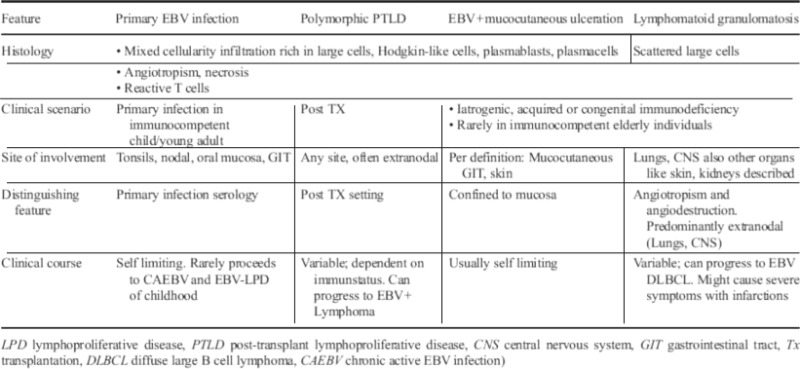
Spectrum of polymorphic EBV driven LPD with variable neoplastic potential

Following the primary infection, patients become asymptomatic virus carriers with a very limited number of EBV-positive B cells. A tissue correlate of this phenomenon is the occasional presence of single small EBV-encoded RNA (EBER)-positive B cells, so called bystander cells, within lymph nodes, mucosa-associated lymphatic tissue, or lungs. Rare complications of primary EBV infection, such as the chronic active EBV (CAEBV) disease and/or EBV-associated hemophagocytic syndrome have recently been reviewed in this journal [3].

## Reactivation of EBV in adults

Since the vast majority of humans have been infected by EBV in childhood and adolescence, EBV-associated LPDs in adults usually arise from reactivation of the virus from EBV-infected memory cells. Such reactivation is generally considered to consist in an escape from the normal immunosurveillance and preferentially occurs at sites representing the physiological reservoir of EBV-infected B cells, such as the lymphatic tissues and mucosa. Therefore, EBV reactivation mainly occurs in immunocompromised individuals. Elderly patients lacking clinically detectable immunosuppression may also suffer from EBV reactivation. In these patients, age-related decline in immunocompetence is assumed to be the cause of EBV-LPD [4, 5]. Certainly, EBV reactivation does occasionally occur in patients lacking objectifiable immunodeficiency. However, the diagnostic pathologist should stress that a clinical work-up of the immune status is warranted once an EBV reactivation is detected.

EBV reactivation in adults occurs primarily in the form of EBV-LPD presenting with a polymorphic histological and clinical picture. Dependent on the clinical scenario and the preferential site of involvement, different types of polymorphic EBV-LPD can be distinguished. They are outlined in Table 2 and include polymorphic posttransplantation lymphoproliferations (PTLD) [6], EBV-positive mucocutaneous ulceration (EBVMCU) [7], and lymphomatoid granulomatosis [8]. Depending on the clinical scenario and the extent of tissue involvement, these conditions cause variable clinical symptoms. Polymorphic EBV-LPD regress in most patients if immunocompetence can be reestablished, e.g., by withholding immunosuppressive therapy. Nevertheless, a subgroup of polymorphic EBV-LPD progress to more aggressive diseases, usually accompanied by a transformation to the morphology of a fully developed lymphoma (designated as “monomorphic” e.g., in the scenario of PTLD).

Polymorphic EBV-associated lymphoproliferations share histomorphological and immunophenotypical features, which are certainly not specific but should alert the histopathologist to an EBV-driven disease when analyzing lymphatic proliferations. These features include the following:

- Tissue necrosis
- Epithelial ulceration
- Angiotropic vasculitis-like lymphoid infiltrates
- Polymorphic picture rich in large cells, Hodgkin-like cells, plasmablasts and plasma cells
- Intermingled large T cells (often CD8 positive)
- B blasts, with weak CD20 expression and expression of CD30

**Fig. 1:**
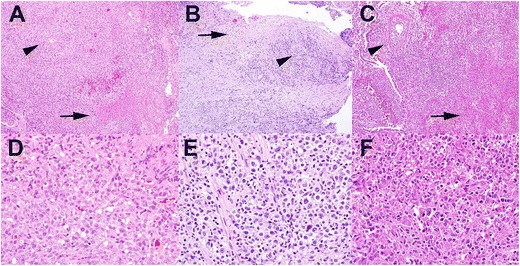
Histomorphology of EBV-associated lymphoproliferative diseases. **a** and **d** illustrates the features of a primary EBV infection in the oral mucosa of a child. As examples of EBV reactivation, an EBV-associated mucocutaneous ulcer in the anal mucosa in a patient treated with azathioprin for myastenia (**b** and **e**) and a lymphomatoid granulomatosis in the lung of a patient (**c** and **f**) are shown. As shared features in all lesions necrosis (*arrow*) and angiotropism (*arrow head*) are indicated. All stainings Hematoxilin and Eosin, original magnification **a**–**c** ×100, **d–f** ×400

If the clinical scenario (immunosuppression) or the histological features are suggestive of an EBV-LPD, testing by EBER in situ hybridization should be initiated (see below). When the diagnosis of an EBV-positive polymorphic LPD has been confirmed by detection of EBV in the tissue, the nomenclature (classification) of the disease is dependent on (i) the clinical context and (ii) the involved site (Table 2). There is considerable overlap of histological and immunophenotypical features between these “entities” (Fig. 1). For example, EBV-LPD histologically resembling a lymphomatoid granulomatosis might occur after organ transplantation, in which case the disorder is named according to both the clinical scenario and the histopathological picture as “EBV-positive polymorphic PTLD under the histopathological picture of a lymphomatoid granulomatosis”. The most important diagnostic message from the pathologist to the treating clinican should be (i) the EBV association of the lesion and (ii) the clear distinction of polymorphic EBV-LPD from fully developed lymphomas.

## EBV-positive lymphomas

EBV-positive lymphomas occur in immunocompetent patients, but are much more frequent in immunocompromised patients. In the latter patient group, the latency type of EBV detectable in the lymphoma cells frequently but not exclusively indicates higher viral activity with expression of more EBV encoded proteins (latency type 3) and sometimes active viral replication. Lymphomas occurring in immunocompetent patients, such as Hodgkin lymphoma, usually display latency types 0/1 or 2 with a more restricted pattern of EBV-associated proteins and no viral replication (Table 3). The diagnostic criteria for these lymphomas have been described elsewhere and do not necessarily require the detection of EBV because of the presence of EBV in the lymphoma cells. In rare cases, detection of EBV is a mandatory diagnostic feature (e.g., in EBV-positive LPD of childhood) [6]. Nevertheless, in certain lymphoma entities, e.g., nasal and extranasal NK T cell lymphoma, angioimmunoblastic T cell lymphoma, primary effusion lymphoma, and plasmablastic lymphoma, EBV association remains an important clue towards the correct diagnosis. Of further relevance might be the identification of EBV-positive diffuse large B cell lymphoma (DLBCL) of the elderly [9]. Histological features that are useful for identifying DLBCL candidates for screening for EBV have been described as angiotropic growth, necrosis, and polymorphic, plasmablastic or Hodgkin-like differentiation and expression of CD30 [6].

**Table 3:** EBV-positive lymphomas and latency types and their association with immunocompromised state

Lineage	Immunocompetence of patients	Entity	Latency type
B cell	Competent	Classical Hodgkin Lymphoma	2
		Endemic Burkitt Lymphoma	0/1
		Sporadic Burkitt Lymphoma	0/1
		EBV + DLBCL of the elderly	Variable
		EBV + DLBCL associated with chronic inflammation (Pyothorax lymphoma)	Predominantly 3
	Compromised	Primary effusion lymphoma	0/1
		Plasmablastic lymphoma	0/1
		Lymphomatoid Granulomatosis Grade 3 and DLBCL arising from the former	3
		Monomorphic PTLD^a^	Variable
		Lymphomas associated with HIV infection^a^	Variable
		Lymphoproliferative disease associated with primary immune disorders^a^	Variable
		Other iatrogenic immunodeficiency-associated lymphoproliferative disorders^a^	3
T cell	Competent	Angioimmunoblastic T cell lymphoma	0/1 or 2
		Extranodal NK/T cell lymphoma	2
	Compromised	EBV-positive T cell lymphoproliferative disease of childhood and young adults^b^	Variable

^a^Diseases under this category are further specified according to histopathology

^b^The immunodeficiency in many of these young patients is postulated but not always objectifiable

*DLBCL* diffuse large B cell lymphoma, *PTLD* posttransplant lymphoproliferative disease

## Distinguishing polymorphic EBV-LPD and EBV-positive lymphoma

The diagnostic criteria for EBV-positive lymphomas are described in the current WHO classification [6]. However, distinguishing polymorphic EBV-LPD and a fully developed EBV-positive lymphoma can be challenging. The most important feature, large sheets of EBV-positive blasts and absence of a polymorphic cellular picture, should raise suspicion of an EBV-positive lymphoma, especially a DLBCL. A specifically difficult task is the differentiation of a polymorphic EBV-LPD from a fully developed classical Hodgkin lymphoma, because the latter may be composed of a similarly polymorphic cellular infiltrate to that of the polymorphic EBV-LPD. Table 4 shows helpful diagnostic features for separating disorders. Pathologists should be aware of the fact that proteins expressed in Hodgkin lymphoma, such as CD15, can also be observed in polymorphic EBV-LPD, like EBVMCU [7], and therefore the final diagnosis will depend not only on the histopathological features but also on the clinical context, including the site of involvement. For example, Hodgkin lymphomas rarely involve epithelial barrier organs, but EBVMCU by definition always do (Table 2). In our experience, clonality testing is not helpful in differentiating EBV-positive lymphoma from polymorphic lymphoproliferations, as viral-driven LPD can also result in a clonal expansion of T and B cells. Testing for EBNA2 expression is useful in some cases, since expression of EBNA2 strongly argues against Hodgkin lymphoma (Table 2).

**Table 4:** Differential diagnostic of polymorphic EBV-positive lymphoproliferative disease (*LPD*) compared to Hodgkin Lymphoma

Feature	Hodgkin Lymphoma	Polymorphic EBV + LPD
Histomorphology	Mixed cellularity infiltration with Hodgkin (−like) cells, necrosis	
	Predominant epitheloid cells and eosinophils	Predominant plasmablasts, plasmacells, angiotropism
Immunophenotype of large cells	CD30 > CD20	CD20 > CD30
Expression of EBV antigens	• EBER + cells equal to LMP1 + cells • EBER + large cells only • EBNA2 negative	• EBER + cells more than LMP1 + cells • EBER + small and large cells • EBNA2 +/−
Clinical scenario	Predominant immunocompetent	Predominant immunocompromised
Site	• Mediastinum • Nodal> > extranodal	• Extranodal> > nodal • Mucosa

## Recommendations for EBV testing

**Fig. 2:**
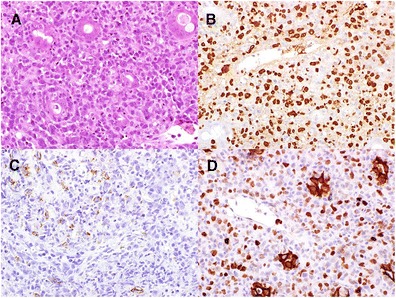
An EBV-associated mucocutaneous ulcer in the colonic mucosa (**a** Hematoxilin and Eosin). Staining pattern of EBV-encoded RNA (EBER, **b**) is nuclear. The protein LMP1 is expressed in the cytoplasm (**c**). EBNA2 protein expression is nuclear (**d**). Note the artificial cytoplasmic staining in the epithelium (**d**). The case illustrates the variability of markers being detectable. EBER usually labels the majority of EBV-infected cells, whereas LMP1 and EBNA2 if positive in the lesion stain only a subset of all EBE-positive cells. Original magnification **a**–**d** ×400

The question as to which specimen should be tested for EBV depends on the histopathological findings on the one hand and on the clinical context on the other hand. Table 3 summarizes our criteria and favored method of testing for EBV, fig. 2 shows typical results. If pathologists are confronted with lymphoid tissue histologically resembling inflammatory (reactive) tissue in immunocompetent patients, the decision whether or not to test for EBV is primarily based on histological features (Table 3). In immunocompromised patients, EBV testing is useful in any histologically inflammatory lesion. The method of testing should be EBER in situ hybridization, which is most sensitive for detecting EBV, especially in lesions that cannot necessarily be expected to express other viral antigens such as LMP1 (Table 3). Since indolent (low grade) B cell lymphomas are virtually never associated with EBV, testing cannot be recommended. For other entities, which are listed in Tables 3 and 5, testing should be part of the standard diagnostic work-up. Immunohistochemical staining for LMP1 is sufficient for Hodgkin lymphoma, since these lymphomas express the latency type 2. However, other lymphomas listed in Table 5, such as diffuse large B cell lymphomas, require testing by EBER because LMP1 is frequently not detectable.

**Table 5:** Recommendations for selecting tissue specimen and methods for EBV testing

Lymphoma	Nodal and extranodal lymphatic tissue not presenting overt lymphoma (polymorphic LPD)	
	Histopathological features	Clinical context
Testing by EBER	Testing by EBER	Testing by EBER
B cell lymphomas		
• Endemic Burkitt Lymhoma	• Hodgkin-like large cells and Hodgkin or Reed-Sternberg cells	• Inherited immunodeficiency
• Plasmablastic lymphoma		
• Primary effusion lymphoma		• Posttransplantation
• Pyothorax associated lymphoma		
• DLBCL with histological features suspicious for EBV association	• Necrosis	• Drug induced immunocompromised state
	• Angiotropism of blastic cells	
• Any monomorphic PTLD		
• Lymphomatoid granulomatosis	• Plasmablastic infiltrate	• HIV infection
T cell lymphomas		
• AILT		
• Extranodal NK/T cell lymphoma		
• EBV-positive T cell lymphoproliferative disease of childhood and young adults		
Testing by EBER or LMP		
• Hodgkin Lymphoma		
Testing by EBER, LMP1 and EBNA2		
• Differential diagnosis between Hodgkin lymphoma and PTLD		

*PTLD* posttransplantation lymphoproliferative disease, *AITL* angioimmunoblastic T cell lymphoma

## Perspective

Although epidemiologic data are lacking, EBV-LPD distinct from overt lymphoma will be increasingly recognized. This is due on the one hand to the aging of many populations, with an increase in age-associated EBV reactivation. On the other hand, widespread access to commercial assays and automatization leads to an increasing use of in situ hybridization for EBER by diagnostic pathologists. Additionally, newly recognized subgroups of lymphomas, such as the EBV-positive DLBCL of the elderly, require the detection of EBV in more lymphoma entities as part of the diagnostic process.
